# Identification of exosomal miR-455-5p and miR-1255a as therapeutic targets for breast cancer

**DOI:** 10.1042/BSR20190303

**Published:** 2020-01-17

**Authors:** Ying Xin, Xueqiang Wang, Kexin Meng, Chao Ni, Zhenye Lv, Dandan Guan

**Affiliations:** 1 Breast and Thyroid Surgical Department, Zhejiang Provincial People’s Hospital of Hangzhou Medical College, Hangzhou City, Zhejiang 310014, China; 2 Department of General Surgery, Yiwu Hospital of Traditional Chinese Medicine, Yiwu, Zhejiang 322000, China

**Keywords:** breast cancers, cell survival, microRNA

## Abstract

Accumulated evidence has demonstrated exosomes of cancer cells carry microRNAs (miRNAs) to non-malignant cells to induce metastasis. The present study aimed to identify crucial exosomal miRNAs for breast cancer (BC) using microarray data (GSE83669 and GSE50429) from Gene Expression Omnibus database, including exosomal samples from human BC cells (MCF7, MDA-MB-231) and normal mammary epithelial cell line (MCF10, MCF-10A), as well as original cell samples. Differentially expressed miRNAs (DEMs) were identified using EdgeR package, and mRNA targets were predicted using miRWalk2 database. The target genes were overlapped with BC genes from Comparative Toxicogenomics Database (CTD) to construct BC-related interaction network. Potential functions were analyzed by DAVID. The expression of crucial miRNAs and target genes were confirmed in other microarray datasets or TCGA sequencing data. Their associations with survival and other clinical characteristics were validated by Kaplan–Meier plotter and LinkedOmics database. As a result, 9 and 8 DEMs were identified to be shared in two datasets for exosomal and original cells, respectively. Further comparison showed that miR-455-5p was specifically differentially expressed in exosomes, and miR-1255a was commonly expressed in exosomal and original cells samples. miR-455-5p could interact with CDKN1B to influence cell cycle process and miR-1255a could regulate SMAD4 to participate in TGF-β signaling pathway. High expressed miR-455-5p (basal-like) and miR-1255a (overall) were associated with poor overall survival, while the high expression of their target genes was associated with excellent overall, recurrence-free or distant metastasis-free survival. In conclusion, the present study preliminarily indicates that exosomal miR-455-5p and miR-1255a may be novel therapeutic targets for BC.

## Introduction

Breast cancer is one of the leading causes of oncological mortality for women [1,2], with an estimated 271,270 new cases and 42,260 cases of deaths in the U.S.A. in 2019 [1]. Although diagnostic methods and therapeutic strategies have been improved, poor prognosis is still present in approximately 30% of patients due to recurrence and metastasis [3,4]. Therefore, it is essential to further understand the molecular mechanisms of breast cancer to develop novel therapeutic modalities.

Recently, it has been widely considered that the development of recurrence and metastasis may be resulted from the interactions between tumor cells and non-malignant cells [5]. Exosomes are membrane-derived nanometer-sized (50–150 nm) vesicles that can be secreted from one kind of cells and then transferred to neighboring or distant cells to influence their phenotypes. Therefore, exosomes may play a critical role in mediating intercellular communication and breast cancer progression [6,7]. Exosomes of cancer cells carry several cargos, among which microRNAs (miRNAs) may be especially important because they could regulate the expression of genes post-transcriptionally in recipient cells by binding to the 3′-untranslated region (3′-UTR) of target messenger RNAs (mRNAs) and leading to their translation inhibition or degradation [8,9]. There has evidence to show the roles of tumor-derived exosomal miRNAs in breast cancer. For example, Wu et al. found that exosomal miR-1246 was highly expressed in breast cancer cell line MDA-MB-231, but extremely low expressed in non-malignant HMLE and MCF-10A cells. Incubation of exosomes from MDA-MB-231 cells with HMLE cells induced a 5-fold increase of intracellular miR-1246 in HMLE cells and enhanced its cell proliferation, migration and chemotherapy resistance ability, but reduced the apoptosis rate. The potential mechanism was to inhibit the expression of its target gene, Cyclin-G2 in HMLE cells [10]. Baroni et al. observed triple-negative cancer-associated fibroblasts secreted miR-9 via exosomes to recipient normal fibroblasts and increased the cell motility of fibroblasts by suppressing the expression of E-cadherin [11]. Di Modica et al. demonstrated that MDA-MB-231 released miR-939 in exosomes can be internalized by endothelial cells and then disrupted the endothelial barrier via down-regulating the expression of VE-cadherin, which is a crucial step for blood vessel invasion and metastasis [12]. Kia et al. identified that treatment with MDA‐MB‐231 cell-derived exosome carrying miR-9 and miR-155 for MCF-7 cells resulted in lower expressions of phosphatase and tensin homologue and dual specificity phosphatase 14 in MCF-7 cells [13]. However, the studies on breast cancer derived exosomal miRNAs remain rare.

There also several studies to investigate the exosomal miRNA expression profiles in order to screen crucial exosomal miRNAs for breast cancer progression. However, most of them focused on chemoresistance mechanisms in breast cancer cells [14,15] or biomarker screening in serum or plasma of patients [16–18]. In the present study, we aimed to identify exosomal differentially expressed miRNAs (DEMs) between breast cancer cells and normal control cells by comprehensive analysis of two microarray datasets downloaded from the public Gene Expression Omnibus (GEO) database. Furthermore, the DEMs of original cells were also screened and compared with exosomal miRNAs to filter exosome-specific, and the common DEMs between exosomes and original cells for breast cancer. These findings may provide novel therapeutic targets for breast cancer.

## Materials and methods

### Data collection

Exosomal miRNA datasets of breast cancer were identified by an electronic search in GEO database (http://www.ncbi.nlm.nih.gov/geo/) using the key word [exosomal (or exosomes) AND breast cancer] on December 2018. The cells or tissues did not undergo any treatment. Four were obtained, including GSE83669, GSE50429, GSE70432 and GSE114329, all of which analyzed the exosomal miRNAs of cell lines. GSE70432 and GSE114329 were excluded due to the lack of normal controls and the lower matching rate, respectively. Thus, only GSE83669 and GSE50429 were used in the following analysis. GSE83669 dataset contained six samples, including exosomal samples of two human breast cancer cells (MCF7 and MDA-MB-231) and one normal mammary epithelial cell line (MCF10) as well as their original cells, which were obtained on Illumina MiSeq platform (GPL15520). GSE50429 consisted of four samples, including exosomal samples of one breast cancer cell (MDA-MB-231) and one normal mammary epithelial cell line (MCF-10A) as well as their original cells, which were sequenced on Illumina Genome Analyzer IIx platform (GPL10999).

### Data preprocessing and identification of DEMs

The raw miRNA read count was normalized using the “normalize.quantiles” function from preprocessCore (version 3.8; http://bioconductor.org/packages/release/bioc/html/preprocessCore.html) in R (version 3.4.1; http://www.R-project.org/). The DEMs between normal mammary epithelial cells and breast cancer cells in exosomal and original cell samples were identified using the EdgeR package (version 3.24.4; http://www.bioconductor.org/packages/release/bioc/html/edgeR.html) [19] of R software. The cut-off values for DEMs were defined as *P* < 0.05 and |log2FC (fold change)| > 1. The heatmap showing the expression pattern of DEMs in samples of two groups was generated using the pheatmap package (version: 1.0.8; https://cran.r-project.org/web/packages/pheatmap) in R based on Euclidean distance. A Venn diagram (http://bioinformatics.psb.ugent.be/webtools/Venn/) was used to visualize the shared DEMs between different datasets of exosomes and original cells as well as the common and specific DEMs between exosomes and original cells.

### Prediction of target genes of DEMs

The target genes of DEMs were predicted using the miRWalk database (version 2.0; http://zmf.umm.uni-heidelberg.de/apps/zmf/mirwalk2/) [20] that included 12 existing miRNA-target prediction programs (miRWalk, MicroT4, miRanda, miRBridge, miRDB, miRMap, miRNAMap, PICTAR2, PITA, RNA22, RNAhybrid and Targetscan). Only the interaction relationships predicted by at least five algorithms were retained according to the threshold value of minimum seed length = 7 at 3′ UTR and *P*-value < 0.05.

### Construction of breast cancer related DEM–mRNAs interaction network

All known breast cancer related genes were downloaded from Comparative Toxicogenomics Database (CTD, http://ctd.mdibl.org/) [21], which was then intersected with the target genes of DEMs. The relationships between the above screened common mRNAs and DEMs were used for constructing potential breast cancer related DEMs–mRNA interaction network that was visualized using Cytoscape software (version 3.4; www.cytoscape.org/) [22].

### Function enrichment analysis

The underlying functions of genes in the miRNA–mRNA interaction network were predicted by searching the online tool of Database for Annotation, Visualization and Integrated Discovery (DAVID) online tool (version 6.8; http://david.abcc.ncifcrf.gov) [23]. The significant Gene ontology (GO) and Kyoto Encyclopedia of Genes and Genomes (KEGG) pathways could be enriched based on the cut-off criterion of *P*-value < 0.05.

### Validation of miRNA and mRNA using TCGA data

The expression of crucial exosomal miRNAs were confirmed in GSE60714 (2 MDA-MB-231 exosomes versus 2 MCF10A exosomes) microarray dataset [24] and miRNAs in original cells were validated in GSE45666 (101 breast tumor versus 15 adjacent breast normal tissue samples) [25] using GEO2R software. The miRNAs and mRNA Seq data of breast cancer (Level 3) were also obtained from The Cancer Genome Atlas (TCGA; https://tcga-data.nci.nih.gov/) to verify the expression levels of crucial miRNAs in original cells and targeted mRNAs. Univariate Cox regression analysis was performed to screen overall survival (OS) related DEMs and genes using the survival package (version 2.4; https://cran.r-project.org/web/packages/survival/index.html), with log-rank < 0.05 as the threshold value. Furthermore, the prognostic significance of miRNAs and mRNAs for breast cancer was also determined by using an online tool, Kaplan–Meier plotter (http://kmplot.com/analysis/) based on the TCGA, METABRIC or microarray data. The associations between miRNAs/mRNAs and other clinical characteristics [such as pathologic_stage, pathology_T_stage, pathology_N_stage, pathology_M_stage, PAM50 (research-based 50-gene prediction analysis of a microarray), estrogen receptor (ER) status, progesterone receptor (PR) status and human epidermal growth factor 2 (HER2)] were examined by searching the online tool, LinkedOmics database (http://www.linkedomics.org/) [26].

## Results

### Differential expression analysis

**Figure 1:**
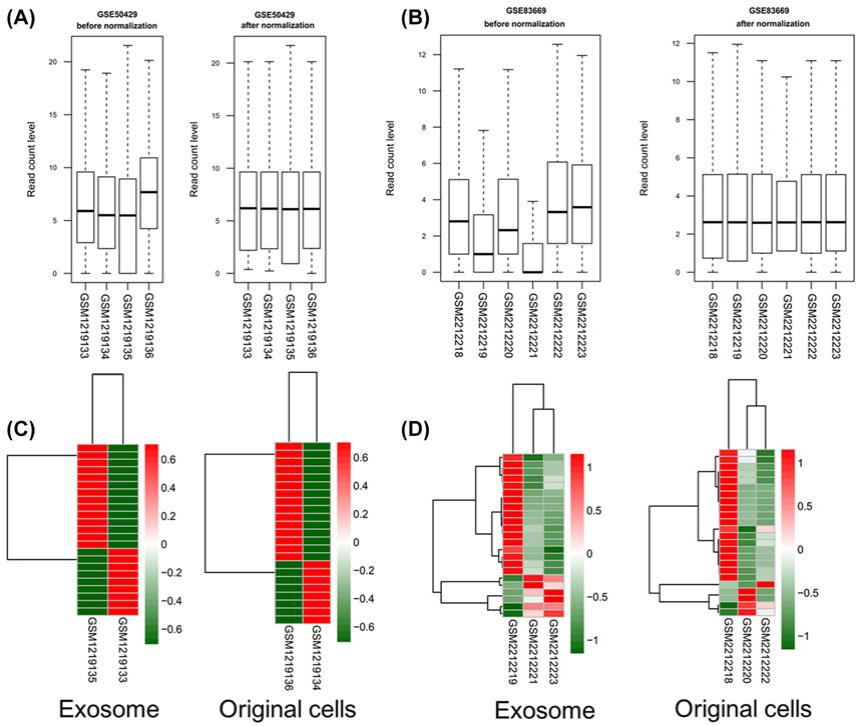
Data processing and identification of differentially expressed miRNAs (**A**) Data processing results before and after normalization of GSE50429. (**B**) Data processing results before and after normalization of GSE83669. (**C**) Heatmap showing differentially expressed miRNAs in exosomal and original cells samples of GSE50429. (**D**) Heatmap showing differentially expressed miRNAs in exosomal and original cells samples of GSE83669. Red and green indicated the high and lower expression, respectively.

**Figure 2:**
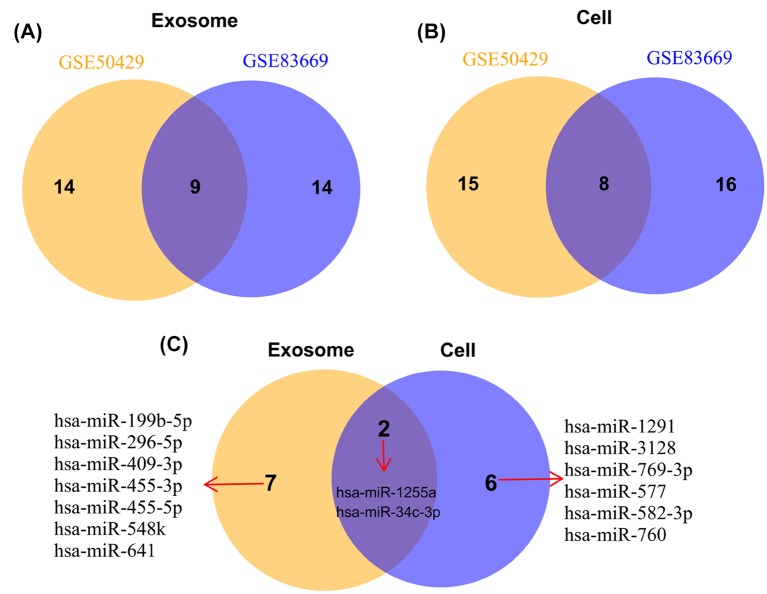
Venn diagram The shared differentially expressed miRNAs between different datasets of exosomes (**A**) and original cells (**B**) as well as the common and specific differentially expressed miRNAs between exosomes and original cells (**C**) were visualized.

After normalization (Figure 1A,B), a total of 23 DEMs between breast cancer and normal cells were respectively identified in the exosomal samples of GSE50429 (Figure 1C) and GSE83669 (Figure 1D) datasets under the thresholds of |logFC| > 1 and *P* < 0.05; while there were 23 and 24 DEMs were identified in the cancer original cells of GSE50429 (Figure 1C) and GSE83669 (Figure 1D) datasets, respectively. After comparison, 9 (Table 1; Figure 2A) and 8 (Table 1; Figure 2B) were found to be shared in the two datasets for exosomal and original cells samples, respectively. Further comparison between the exosomal and original cells indicated that 7 were specifically differentially expressed in exosomes, 6 were specific for original cells and 2 were common for exosomal and original cells (Figure 2C).

**Table 1:** Common differentially expressed miRNAs in two datasets

	GSE83669			GSE50429		
	ID	logFC	*P*-value	ID	logFC	*P*-value
Exosomes	hsa-miR-1255a	−1.75	5.36E-03	hsa-miR-1255a	1.22	4.90E-02
	hsa-miR-199b-5p	3.03	1.91E-03	hsa-miR-199b-5p	4.74	4.90E-02
	hsa-miR-296-5p	−3.42	3.06E-03	hsa-miR-296-5p	-3.03	4.46E-03
	hsa-miR-34c-3p	1.22	4.90E-02	hsa-miR-34c-3p	-2.79	6.8E-03
	hsa-miR-409-3p	1.16	1.31E-02	hsa-miR-409-3p	3.31	4.01E-02
	hsa-miR-455-3p	2.72	3.45E-03	hsa-miR-455-3p	6.65	4.90E-02
	hsa-miR-455-5p	3.14	1.05E-03	hsa-miR-455-5p	6.03	4.90E-02
	hsa-miR-548k	−4.13	7.66E-04	hsa-miR-548k	-1.12	1.77E-03
	hsa-miR-641	−1.33	4.90E-02	hsa-miR-641	1.50	4.90E-02
Original	hsa-miR-1255a	1.09	3.07E-02	hsa-miR-1255a	1.33	2.89E-02
	hsa-miR-1291	4.09	4.26E-02	hsa-miR-1291	1.42	2.89E-02
	hsa-miR-3128	1.09	3.07E-02	hsa-miR-3128	2.54	2.19E-02
	hsa-miR-34c-3p	1.43	3.27E-02	hsa-miR-34c-3p	-4.59	3.13E-03
	hsa-miR-577	−1.64	1.94E-02	hsa-miR-577	1.02	4.24E-02
	hsa-miR-582-3p	−4.00	2.58E-03	hsa-miR-582-3p	1.63	1.46E-02
	hsa-miR-760	−1.32	1.92E-02	hsa-miR-760	1.27	3.44E-02
	hsa-miR-769-3p	33.42	4.90E-02	hsa-miR-769-3p	1.43	2.27E-02

Abbreviation: FC, fold change.

### DEM–mRNAs interaction network

**Figure 3:**
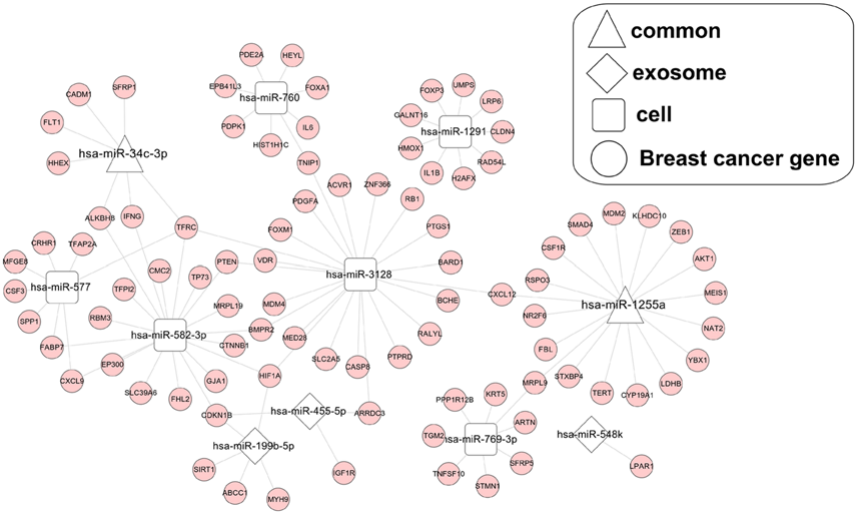
Breast cancer related miRNA-mRNA interaction network

The target genes of the 15 DEMs in Figure 2C were predicted using the miRwalk 2.0 database, with 4234 interaction relationships obtained. By overlapping with 550 breast cancer related genes downloaded from the CTD, 91 target genes for 11 (exosomal specific: 3; original cell specific: 6; common: 2) DEMs were extracted to construct the breast cancer associated interaction network (Figure 3). In this network, hsa-miR-455-5p, which was exosomal specific and expressed consistently in two datasets (up-regulated), could interact with CDKN1B (cyclin dependent kinase inhibitor 1B); although miR-1255a was commonly DEM in exosomal and original cells, its expression (up-regulated) was only consistent in original cells of two datasets, but only up-regulated for exosomal samples of GSE83669 dataset. miR-1255a was predicted to interact with SMAD4 (SMAD family member 4).

### Function enrichment for target genes of DEMs

**Figure 4:**
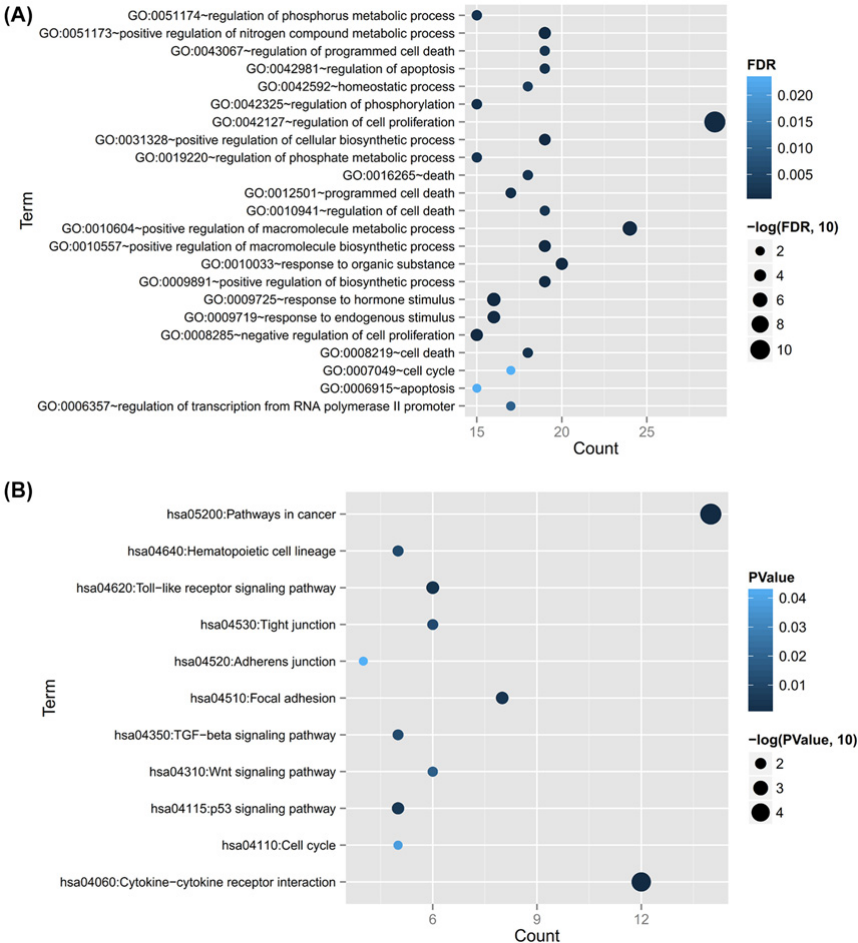
Function enrichment analyses for the target genes of differentially expressed miRNAs (**A**) GO enrichment results. (**B**) KEGG pathway enrichment results. GO, Gene ontology; KEGG, Kyoto Encyclopedia of Genes and Genomes; FDR, false discovery rate.

GO terms and KEGG pathway enrichment analyses were performed for the target genes in the Figure 3 to predict their potential functions by using DAVID software. As a result, 23 GO biology process terms were enriched for all the target genes of DEMs, mainly involving GO:0042127∼regulation of cell proliferation (CDKN1B, SMAD4) and GO:0007049∼cell cycle (CDKN1B) (Table 2; Figure 4). Furthermore, 11 KEGG pathways were also enriched, such as hsa05200:Pathways in cancer (CDKN1B, SMAD4), hsa04350:TGF-β signaling pathway (SMAD4) and hsa04110:Cell cycle (CDKN1B, SMAD4) (Table 2; Figure 4).

**Table 2:** Function enrichment for mRNAs in the miRNA–mRNA interaction network

Category	Term	*P*-value	Genes
GO	GO:0042127∼regulation of cell proliferation	5.38E-15	CSF3, PDGFA, FOXM1, PTGS1, BMPR2, GJA1, ZEB1, PTEN, CTNNB1, VDR, IGF1R, KRT5, HMOX1, IFNG, TGM2, IL1B, IL6, FLT1, SMAD4, MFGE8, RB1, FOXP3, SIRT1, HHEX, CDKN1B, HIF1A, MDM2, MDM4, FABP7
	GO:0010604∼positive regulation of macromolecule metabolic process	7.60E-10	IL6, PDGFA, FOXA1, BMPR2, SMAD4, FHL2, GJA1, RB1, ZEB1, FOXP3, TP73, CTNNB1, AKT1, HHEX, IGF1R, EP300, HIF1A, HMOX1, IFNG, MDM2, IL1B, H2AFX, BARD1, ACVR1
	GO:0009725∼response to hormone stimulus	4.56E-09	IL6, PDGFA, PTGS1, FHL2, MFGE8, STXBP4, PTEN, CTNNB1, AKT1, IGF1R, PDPK1, EP300, BCHE, HMOX1, IL1B, SPP1
	GO:0009719∼response to endogenous stimulus	1.72E-08	IL6, PDGFA, PTGS1, FHL2, MFGE8, STXBP4, PTEN, CTNNB1, AKT1, IGF1R, PDPK1, EP300, BCHE, HMOX1, IL1B, SPP1
	GO:0008285∼negative regulation of cell proliferation	3.07E-08	IL6, SMAD4, GJA1, RB1, ZEB1, FOXP3, PTEN, VDR, CDKN1B, KRT5, HMOX1, IFNG, IL1B, MDM4, FABP7
	GO:0051173∼positive regulation of nitrogen compound metabolic process	4.18E-08	IL6, PDGFA, FOXA1, SMAD4, FHL2, RB1, ZEB1, FOXP3, TP73, CTNNB1, AKT1, HHEX, IGF1R, EP300, HIF1A, IFNG, IL1B, H2AFX, ACVR1
	GO:0010033∼response to organic substance	4.18E-08	IL6, PDGFA, RBM3, PTGS1, FHL2, MFGE8, STXBP4, PTEN, CTNNB1, AKT1, IGF1R, PDPK1, EP300, TFRC, BCHE, HMOX1, IFNG, CASP8, IL1B, SPP1
	GO:0010557∼positive regulation of macromolecule biosynthetic process	5.28E-08	IL6, PDGFA, FOXA1, SMAD4, FHL2, RB1, ZEB1, FOXP3, TP73, CTNNB1, AKT1, HHEX, IGF1R, EP300, HIF1A, HMOX1, IFNG, IL1B, ACVR1
	GO:0031328∼positive regulation of cellular biosynthetic process	1.06E-07	IL6, PDGFA, FOXA1, SMAD4, FHL2, RB1, ZEB1, FOXP3, TP73, CTNNB1, AKT1, HHEX, IGF1R, EP300, HIF1A, HMOX1, IFNG, IL1B, ACVR1
	GO:0009891∼positive regulation of biosynthetic process	1.32E-07	IL6, PDGFA, FOXA1, SMAD4, FHL2, RB1, ZEB1, FOXP3, TP73, CTNNB1, AKT1, HHEX, IGF1R, EP300, HIF1A, HMOX1, IFNG, IL1B, ACVR1
	GO:0012501∼programmed cell death	6.40E-07	IL6, CADM1, GJA1, SIRT1, PTEN, TP73, CTNNB1, AKT1, SFRP5, TNFSF10, EP300, CDKN1B, IFNG, CASP8, TGM2, IL1B, MDM4
	GO:0042325∼regulation of phosphorylation	7.01E-07	IL6, FLT1, PDGFA, BMPR2, SMAD4, RB1, LPAR1, PTEN, TP73, AKT1, PDPK1, CDKN1B, IFNG, IL1B, BARD1
	GO:0008219∼cell death	1.12E-06	IL6, CADM1, GJA1, SIRT1, PTEN, TP73, CTNNB1, AKT1, SFRP5, TNFSF10, EP300, CDKN1B, HMOX1, IFNG, CASP8, TGM2, IL1B, MDM4
	GO:0042981∼regulation of apoptosis	1.13E-06	IL6, CADM1, SIRT1, PTEN, TP73, AKT1, VDR, IGF1R, TNFSF10, CDKN1B, SFRP1, HMOX1, IFNG, CASP8, TGM2, IL1B, TERT, BARD1, ACVR1
	GO:0051174∼regulation of phosphorus metabolic process	1.13E-06	IL6, FLT1, PDGFA, BMPR2, SMAD4, RB1, LPAR1, PTEN, TP73, AKT1, PDPK1, CDKN1B, IFNG, IL1B, BARD1
	GO:0019220∼regulation of phosphate metabolic process	1.13E-06	IL6, FLT1, PDGFA, BMPR2, SMAD4, RB1, LPAR1, PTEN, TP73, AKT1, PDPK1, CDKN1B, IFNG, IL1B, BARD1
	GO:0016265∼death	1.23E-06	IL6, CADM1, GJA1, SIRT1, PTEN, TP73, CTNNB1, AKT1, SFRP5, TNFSF10, EP300, CDKN1B, HMOX1, IFNG, CASP8, TGM2, IL1B, MDM4
	GO:0043067∼regulation of programmed cell death	1.30E-06	IL6, CADM1, SIRT1, PTEN, TP73, AKT1, VDR, IGF1R, TNFSF10, CDKN1B, SFRP1, HMOX1, IFNG, CASP8, TGM2, IL1B, TERT, BARD1, ACVR1
	GO:0010941∼regulation of cell death	1.37E-06	IL6, CADM1, SIRT1, PTEN, TP73, AKT1, VDR, IGF1R, TNFSF10, CDKN1B, SFRP1, HMOX1, IFNG, CASP8, TGM2, IL1B, TERT, BARD1, ACVR1
	GO:0042592∼homeostatic process	2.04E-06	IL6, FOXA1, RB1, LPAR1, FOXP3, CXCL12, CTNNB1, AKT1, VDR, EP300, HIF1A, TFRC, HMOX1, IFNG, TGM2, IL1B, TERT, BARD1
	GO:0006357∼regulation of transcription from RNA polymerase II promoter	6.13E-06	IL6, FOXA1, SMAD4, FHL2, RB1, ZEB1, FOXP3, SIRT1, CTNNB1, VDR, HHEX, EP300, HIF1A, HMOX1, MDM2, TFAP2A, MDM4
	GO:0006915∼apoptosis	1.37E-05	IL6, CADM1, GJA1, SIRT1, PTEN, TP73, CTNNB1, AKT1, SFRP5, TNFSF10, EP300, IFNG, CASP8, IL1B, MDM4
	GO:0007049∼cell cycle	1.39E-05	FOXM1, RB1, MYH9, RAD54L, TP73, CTNNB1, AKT1, HHEX, EP300, CDKN1B, IFNG, MDM2, H2AFX, MDM4, STMN1, BARD1, ACVR1
KEGG pathway	hsa05200:Pathways in cancer	1.68E-05	IL6, PDGFA, SMAD4, RB1, PTEN, CTNNB1, AKT1, IGF1R, EP300, HIF1A, CDKN1B, CASP8, MDM2, CSF1R
	hsa04060:Cytokine–cytokine receptor interaction	5.04E-05	CSF3, TNFSF10, IL6, FLT1, PDGFA, IFNG, BMPR2, CXCL9, IL1B, CXCL12, CSF1R, ACVR1
	hsa04620:Toll-like receptor signaling pathway	3.47E-03	AKT1, IL6, CASP8, CXCL9, IL1B, SPP1
	hsa04510:Focal adhesion	4.01E-03	AKT1, IGF1R, PDPK1, FLT1, PDGFA, PTEN, CTNNB1, SPP1
	hsa04115:p53 signaling pathway	4.89E-03	CASP8, MDM2, MDM4, PTEN, TP73
	hsa04640:Hematopoietic cell lineage	1.11E-02	CSF3, IL6, TFRC, IL1B, CSF1R
	hsa04530:Tight junction	1.14E-02	AKT1, EPB41L3, CLDN4, MYH9, PTEN, CTNNB1
	hsa04350:TGF-β signaling pathway	1.16E-02	EP300, IFNG, SMAD4, BMPR2, ACVR1
	hsa04310:Wnt signaling pathway	1.83E-02	SFRP5, EP300, SFRP1, SMAD4, LRP6, CTNNB1
	hsa04110:Cell cycle	3.80E-02	CDKN1B, EP300, SMAD4, MDM2, RB1
	hsa04520:Adherens junction	4.34E-02	IGF1R, EP300, SMAD4, CTNNB1

Abbreviations: GO, Gene ontology; KEGG, Kyoto Encyclopedia of Genes and Genomes.

### Validation of crucial DEMs and mRNAs using another datasets

miR-1255a and miR-455-5p were also found to be up-regulated in MDA-MB231 and MCF7 exosomes compared with MCF10A exosomes in GSE60714 dataset and the study of Melo et al. [24] (Table 3).

**Table 3:** Confirmation of expressions of crucial miRNAs and mRNAs using other datasets

Dataset	Symbol	Tumor	Control	logFC	*P*-value
Melo et al. (Exosome: MCF7 versus MCF10A) [24]	miR-1255a	2.09	1.80		
	miR-455-5p	3.05	2.97		
GSE60714 (Exosome, MDA-MB231 versus MCF10A) [24]	miR-1255a			2.14	0.02
	miR-455-5p			3.19	0.02
GSE45666 (tissue)	miR-1255a			5.27	7.14E-03
TCGA (tissue)	miR-1255a	0.80 ± 0.73	0.67 ± 0.51		0.509
	miR-455	6.93 ± 1.19	6.80 ± 0.64		<0.001
	SMAD4	10.47± 0.53	11.13±0.27		<0.001
	CDKN1B (basal-like)	43.84 (11.39-81.35)	63.55 (36.69-104.94)		<0.001

Abbreviations: CDKN1B, cyclin dependent kinase inhibitor 1B; FC, fold change; SMAD4 (SMAD family member 4); TCGA, The Cancer Genome Atlas.

**Figure 5:**
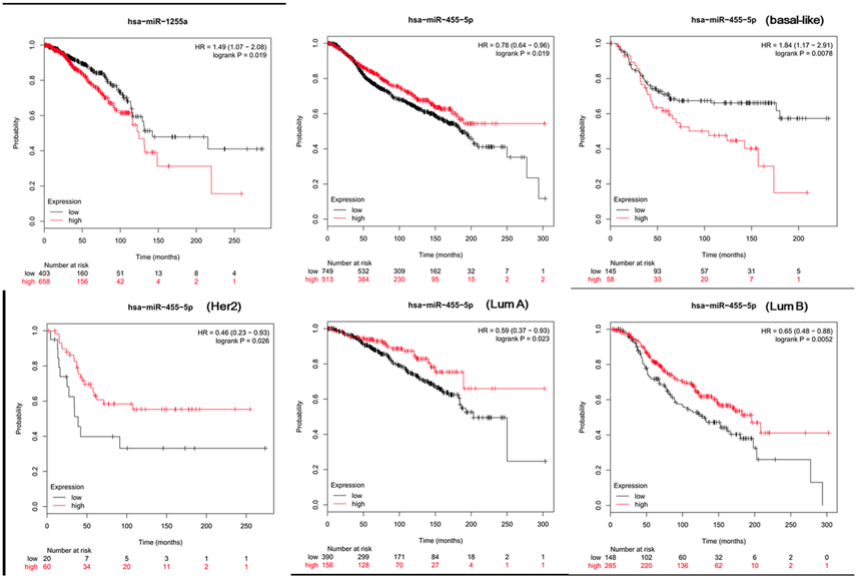
Kaplan–Meier Plotter analysis to display the correlation of differentially expressed miRNAs with survival for patients with breast cancer TCGA data were used for miR-1255a. METABRIC data were used for miR-455-5p. Overall survival was analyzed; HR, hazard ratio; METABRIC, Molecular Taxonomy of Breast Cancer International Consortium; TCGA, The Cancer Genome Atlas.

**Figure 6:**
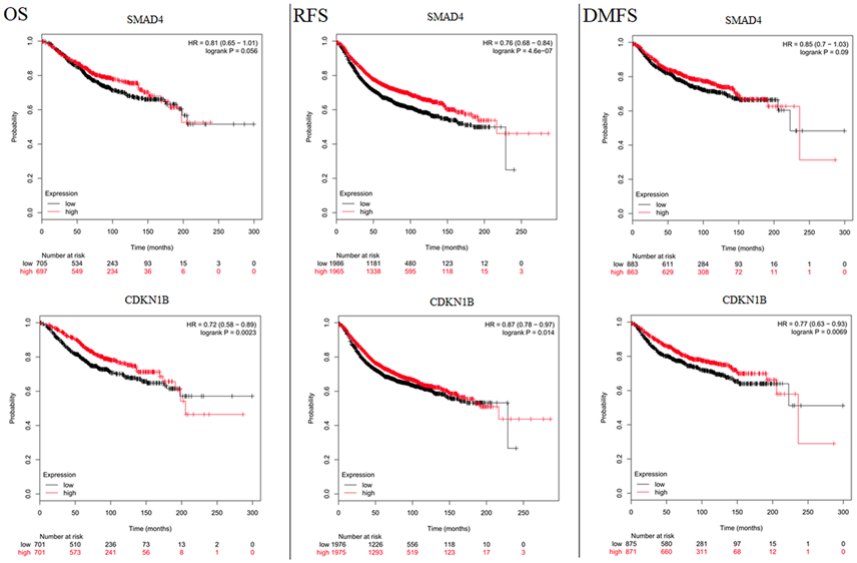
Kaplan–Meier Plotter analysis to display the correlation of target genes of miRNAs with survival for patients with breast cancer Microarray data were used for genes; DMFS, distant metastasis-free survival; HR, hazard ratio; OS, overall survival; RFS, recurrence-free survival.

**Figure 7:**
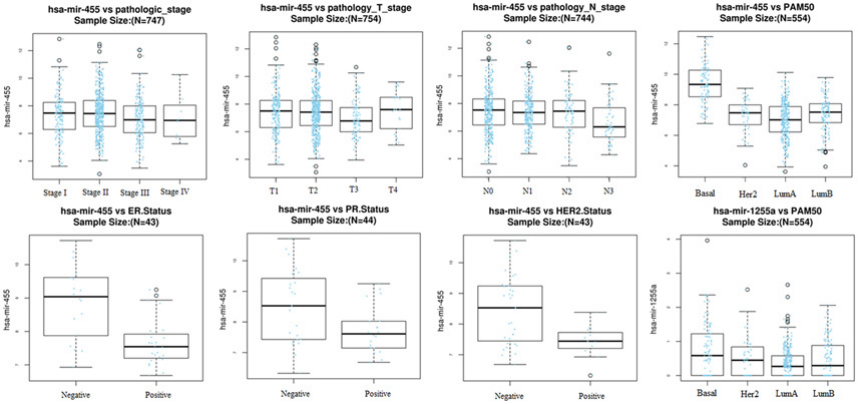
The associations between differentially expressed miRNAs and other clinical characteristics LinkedOmics database with TCGA data was used for this analysis; ER, estrogen receptor; HER2, human epidermal growth factor 2; PAM50, research-based 50-gene prediction analysis of a microarray; PR, progesterone receptor; TCGA, The Cancer Genome Atlas.

**Figure 8:**
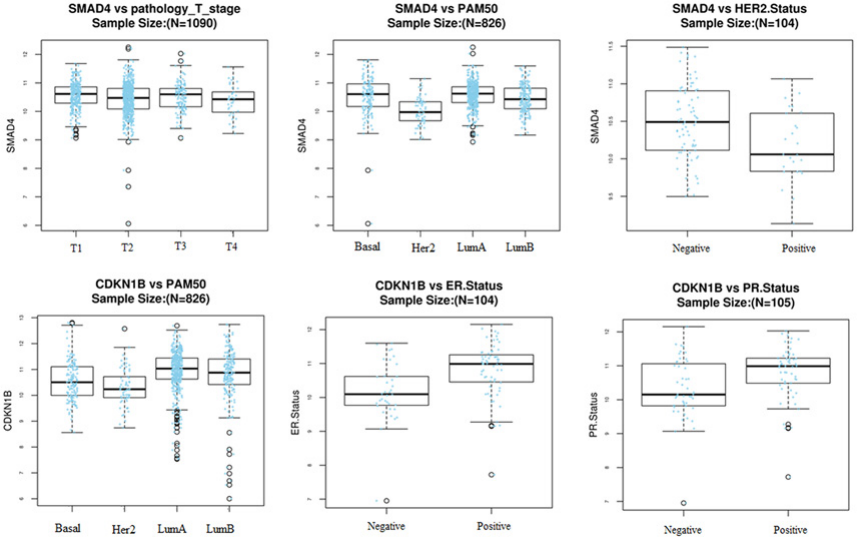
The associations between target genes of miRNAs and other clinical characteristics LinkedOmics database with TCGA data was used for this analysis; ER, estrogen receptor; HER2, human epidermal growth factor 2; PAM50, research-based 50-gene prediction analysis of a microarray; PR, progesterone receptor; TCGA, The Cancer Genome Atlas.

A total of 1180 miRNA–mRNA matched samples of breast cancer (including 1176 cancer and 104 normal tissues) were collected from the TCGA and the expression levels of crucial DEMs (miR-1255a and miR-455) and their target genes (SMAD4 and CDKN1B) were calculated. In line with our expected, miR-455 was found to be significantly up-regulated, but SMAD4 and CDKN1B were significantly down-regulated in cancer (Table 3). Although miR-1255a was also relatively higher in the cancer tissues compared with control, no statistical difference was observed (Table 3). Thus, GSE45666 was also used to further explore the expression of miR-1255a in breast cancer tissues, the results of which indicated miR-1255a was significantly higher expressed (Table 3). Univariate Cox regression analysis was used to explore whether they were associated with the OS using the TCGA data; however, no significant results were detected (data not shown). In order to further confirm their associations with prognosis, Kaplan–Meier plotter database was used. The results showed high expressed has-miR-1255a was a risk factor for poor prognosis (Figure 5). High expression of CDKN1B was associated with excellent OS, recurrence-free survival (RFS) and distant metastasis-free survival (DMFS); SMAD4 was associated with RFS, but not OS and DMFS (Figure 6). miR-455-5p seemed to be a protective factor for excellent prognosis (Figure 5), which was not consistent with our expected. Subsequently, the associations between the above miRNAs/mRNAs and other clinical characteristics were also analyzed. As a result, miR-455 and SMAD4 were associated with Pathologic_T stage; miR-455 was with Pathologic_N stage; all genes and miRNAs were related with PAM50; miR-455 was also correlated with ER status, PR status and HER2 status; CDKN1B was correlated with ER status and PR status; there was an association between HER2 status and SMAD4 (Table 4; Figures 7 and 8). From the result of Figure 7, we could see that the miR-455 was especially highly expressed in basal-like subtype and thus, we further investigated the prognosis association of miR-455-5p with various subtypes of breast cancer. As anticipated, the results showed that up-regulated miR-455-5p predicted poor prognosis in basal-like subtype (Figure 5).

**Table 4:** Clinical association of differentially expressed miRNAs and their target genes

Query	*P*-value							
	miR-1255a	miR-1291	miR-455	miR-199b	IL1B	SMAD4	FOXP3	CDKN1B
Pathologic_stage (Kruskal–Wallis Test)	3.98E-01	6.00E-01	**2.60E-02**	2.83E-01	**2.64E-04**	1.01E-01	8.08E-01	7.92E-01
Pathology_T_stage (Kruskal–Wallis Test)	2.90E-01	3.48E-01	**2.58E-02**	**3.67E-03**	**1.22E-05**	**4.17E-03**	**6.95E-03**	1.52E-01
Pathology_N_stage (Kruskal–Wallis Test)	2.43E-01	1.48E-01	**1.10E-03**	**3.26E-02**	**5.96E-03**	1.48E-01	4.50E-01	5.46E-01
Pathology_M_stage (Wilcox Test)	6.95E-01	3.14E-01	4.37E-01	6.35E-01	**4.26E-02**	6.08E-01	3.68E-01	5.55E-01
PAM50 (Kruskal–Wallis Test)	**5.17E-04**	**9.84E-03**	**2.76E-35**	3.71E-01	**1.51E-07**	**2.77E-14**	**1.23E-16**	**1.12E-15**
ER.Status (Wilcox Test)	1.38E-01	2.36E-01	**3.83E-04**	7.34E-01	2.16E-01	5.34E-01	**1.50E-02**	**2.64E-04**
PR.Status (Wilcox Test)	2.05E-01	3.23E-01	**3.42E-02**	3.52E-01	3.94E-01	2.46E-01	**9.99E-03**	**2.38E-03**
HER2.Status (Wilcox Test)	2.10E-01	5.18E-01	**1.23E-02**	**7.81E-03**	3.78E-01	**7.96E-03**	**3.00E-02**	6.23E-02
Radiation_therapy (Wilcox Test)	1.15E-01	8.01E-01	9.82E-01	1.11E-01	7.37E-01	3.28E-01	6.12E-02	4.98E-01

Abbreviations: CDKN1B, cyclin dependent kinase inhibitor 1B; ER, estrogen receptor; HER2, human epidermal growth factor 2; PAM50, research-based 50-gene prediction analysis of a microarray; PR, progesterone receptor; SMAD4, SMAD family member 4. Bold indicated the statistical significance (*P* < 0.05).

## Discussion

In the present study, we identified two important exosomal miRNAs for breast cancer, including miR-455-5p and miR-1255a. High expressed miR-455-5p may exert tumor promoting roles by inhibiting the expression of CDKN1B and influencing cell cycle, which led to poor prognosis in basal-like subtype (i.e. triple-negative breast cancer, TNBC). High expressed miR-1255a may be oncogenic by down-regulating SMAD4 and affecting TGF-β signaling pathway, which resulted in poor prognosis overall.

Although the roles of miR-455-5p in exosomes have not been investigated previously, its tumor-promoting functions in original cancer cells may indirectly verify our results because of the similar expression trend confirmed using the TCGA data. For example, the study of Aili et al. showed miR-455-5p was significantly up-regulated in breast cancer tissues. High expressed miR-455-5p was an independent prognostic factor for poor survival for breast cancer patients. *In vitro* experiments showed overexpression of miR-455-5p accelerated invasiveness and migration capabilities of breast cancer cells [27]. Li et al. also found that miR-455 was intensively overexpressed in TNBC tissue and cells (MDA-MB-231 and MDA-MB-468) compared with the hormone receptor positive tissues and cells (MCF-7). Functional assays showed that miR-455-3p enhanced cell proliferative, invasive and migratory abilities in TNBC cell lines [28]. Furthermore, studies on non-small cell lung cancer [29], colon cancer [30] and oral squamous cancer cells [31] also verified that miR-455-5p may function as a potential oncogene. In addition, our study also showed exosomal miR-455-5p in breast cancer cells may influence the phenotype of neighboring or distant non-malignant recipient cells by decreasing the expression of CDKN1B gene. CDKN1B, also known as p27Kip1, is a cyclin-dependent kinase inhibitor and thus is a negative cell-cycle regulator. CDKN1B had been observed to be significantly down-regulated in breast cancer tissues and associated with increasing tumor grade, mitosis, poor overall and disease-free cancer survival [32,33]. These findings were also confirmed in our study. Up-regulation of CDKN1B significantly inhibited proliferation, invasion, caused cell cycle arrest in G1 and induced apoptosis of human breast cancer cells [34,35]. Therefore, exosomal miR-455-5p in breast cancer cells may trigger the malignant phenotype of recipient cells via regulating CDKN1B gene; however, the interaction of miR-455-5p and CDKN1B has not been reported previously and needs further confirmation.

miR-1255a is a shared DEM in exosomes and original cells, indicating its important roles as a therapeutic target for breast cancer. However, the expression and functions of miR-1255a in exosomes and cancer original cells have rarely been reported, except occasional identification of another miR-1255 member miR-1255b in cancer. For example, the study of Tölle et al. showed that miR-1255b-5p was significantly increased in the urine of patients with invasive bladder cancer compared with the control group. The urine miR-1255b-5p reached 68% specificity and 85% sensitivity in the diagnosis of invasive bladder cancer [36]. Choi et al. demonstrated that the inhibition of miR-1255b increased the expression of BRCA1 in breast cancer and ovarian cancer cells [37]. Decreased expression of BRCA1 accelerated the growth of malignant mammary cells [38] and was associated with high-grade, advanced lymph node stage, larger size and vascular invasion in breast cancer [39]. High expression of BRCA1 was related with better survival for the breast cancer patients [40]. In line with these studies, we also found miR-1255a was up-regulated in breast cancer cells and tissues and correlated with poor prognosis. More importantly, we predicted miR-1255a may regulate the expression of SMAD4 in breast cancer cells. Accumulating evidence has proved that SMAD4 is a key downstream effector of transforming growth factor-β (TGF-β) signaling pathway, which is known to reduce breast cancer cell invasion and tumor-induced angiogenesis [41,42]. Zhang et al. reported that the expression of SMAD4 and TGF-β receptor can be decreased by TGF-β1 stimulation and promoted the migration and invasion of hepatocellular carcinoma cells [43]. Thus, SMAD4 may also be down-regulated in breast cells and tissues, which has been validated in studies of Stuelten et al. [44] and Zhong et al. [45]. In line with these studies, we also found that SMAD4 has significantly lower expression in breast tissues using TCGA data and its lower expression was associated with poor RFS. Accordingly, exosomal and non-exosomal miR-1255 may be involved in the malignant phenotype of breast cancer cells or recipient cells via regulating TGF-β receptor-SMAD4 pathways; however, the interaction relationship between miR-1255a and SMAD4 needs further confirmation.

The present study had certain limitations: First, sample size and the number of cell lines used for identification of exosomal miRNAs were relatively small, which may be the potential cause to lead to some inconsistent conclusions in different datasets. Although there were six breast cancer cell lines that were included in GSE114329 dataset, the matching rate was very low and most of the expressions of mRNAs were zero and it could not be analyzed. Second, the TCGA data did not include the exosomal miRNAs in breast cancer tissues and the associations with clinical characteristics were only the results preliminarily predicted according to their expression in original cells. Third, although our study preliminarily detected the opposite expression between miRNAs and their target genes, *in vitro* and *in vivo* experiments (RNA-binding protein immunoprecipitation, luciferase receptor analysis, knockout or overexpression) are necessary to confirm their interaction. Fourth, the exosomal mechanisms of the identified miRNAs also need wet experiments (exosome inhibitor GW4869, non-malignant cell co-culture) to validate.

In conclusion, the present study preliminarily indicates that exosomal miR-455-5p and miR-1255a may be novel therapeutic targets for breast cancer. They may be transferred from the breast cancer cells to non-malignant recipient cells to inhibit the expression of CDKN1B and SMAD4 and result in poor prognosis of patients.

## Abbreviations

3′-UTR: 3′-untranslated region
CDKN1B: cyclin dependent kinase inhibitor 1B
CTD: Comparative Toxicogenomics
DAVID: Database for Annotation, Visualization and Integrated Discovery
DEM: differentially expressed miRNA
DMFS: distant metastasis-free survival
ER: estrogen receptor
FC: fold change
GEO: Gene Expression Omnibus
GO: Gene ontology
HER2: human epidermal growth factor 2
KEGG: Kyoto Encyclopedia of Genes and Genomes
microRNA: miRNA
mRNA: messenger RNA
OS: overall survival
PAM50: research-based 50-gene prediction analysis of a microarray
PR: progesterone receptor
RFS: recurrence-free survival
SMAD4: (SMAD family member 4)
TCGA: The Cancer Genome Atlas
TGF-β: transforming growth factor-β
